# The complete chloroplast genome sequence of *Potentilla tanacetifolia* Willd. ex Schltdl.

**DOI:** 10.1080/23802359.2021.1872436

**Published:** 2021-02-09

**Authors:** Yang Li, Xiang Li, Fangbing Ding, Qian Li, Huiling Yan, Renna Li, Lei Zhang, Hong Wang, Yu Zhao

**Affiliations:** a Xi’an Botanical Garden of Shaanxi Province, Institute of Botany of Shaanxi Province, Xi’an, China; b Shaanxi Engineering Research Centre for Conservation and Utilization of Botanical Resources, Xi’an, China; c School of Electronic and Information, Shaanxi Institute of Technology, Xi’an, China

**Keywords:** *Potentilla tanacetifolia*, chloroplast genome, phylogenetic analysis

## Abstract

*Potentilla tanacetifolia* Willd. ex Schltdl. is a perennial herb in China, which has high ecological and economic values. Its complete chloroplast genome was reported in this study for the first time. The whole chloroplast genome was 157, 051 base pairs in length with 129 genes, including 84 protein-coding genes, 37 tRNAs, and 8 rRNAs. In addition, phylogenetic analysis showed a sister relationship between *P. tanacetifolia* and *P. chinensis*.

*Potentilla tanacetifolia* Willd. ex Schltdl. is a perennial herb as a member of the *Potentilla* belonged to the family Rosaceae and widely distributed in China, which has persisted largely in an undomesticated state that is highly resistant to different environmental stresses (Li et al. 2019). *P. tanacetifolia* has high ecological and economic values, that seedlings of *P. tanacetifolia* in spring is one of important fodder plant. Furthermore, *P. tanacetifolia* is usually regarded as an indicator plant for community succession because of its sensitivity to the environment change and grazing (Li et al. 2011). It makes that *P. tanacetifolia* represents an excellent model for understanding how different evolutionary forces have sculpted the variation patterns in the genome during the process of population differentiation and ecological speciation, because *P. tanacetifolia* has wide geographic distribution, and adaptability to different environments. In the present research, we characterized the whole chloroplast genome of *P. tanacetifolia* and comprehended more about genetic information of this species, which can contribute to the understanding population genetics studies of *P. tanacetifolia*.

The fresh leaves of *P. tanacetifolia* were collected from Duolun County (42°02′N, 116°17′E, 1324 m asl), Inner Mongolia Autonomous Region of China. The leaf samples were silica-dried and taken to the laboratory until DNA extraction. The voucher specimen (HF2019002) was laid in the Herbarium of Xi’an Botanical Garden of Shaanxi Province, China. The total genomic DNA was isolated according to a modified CTAB method (Doyle and Doyle 1987). Total genome DNA of *P. tanacetifolia* was sequenced by Illumina Hiseq 2500 Sequencing System (Illumina, Hayward, CA) to construct the shotgun library. The qualified clean reads were assembled by NOVOPlasty (Dierckxsens et al. 2017), with *P. stolonifera* (NC_044418) as a reference. The low-quality sequences were filtered out using CLC Genomics Workbench v8.0 (CLC Bio, Aarhus, Denmark). The complete chloroplast genome of *P. tanacetifolia* was annotated by Geneious ver. 10.1 (http://www.geneious.com, Kearse et al. 2012) and online program Chloroplast Genome Annotation, Visualization, Analysis, and GenBank Submission (CPGAVAS) (Institute of Medicinal Plant development, Chinese Academy of Medical Sciences and Peking Union Medical College, Beijing, China) (Zuo et al. 2017). Finally, the validated complete chloroplast genome of *P. tanacetifolia* was deposited in Genbank (Accession number MW125592).

The complete chloroplast genome of *P. tanacetifolia* was 157, 051 bp in length, containing a large single-copy region (LSC) of 86, 147 bp, a small single-copy region (SSC) of 18, 883 bp, and two inverted repeat (IR) regions of 26, 010 bp. The overall GC content is 36.8%. The genome contains 129 complete genes, including 84 protein-coding genes, 37 tRNA genes and 8 rRNA genes.

To identify the phylogenetic position of *P. tanacetifolia*, 8 complete chloroplast genomes sequence of *Potentilla* were obtained from NCBI GenBank, and *Agrimonia pilosa* and *Sanguisorba officinalis* was used as out-group for phylogenetic analysis. The 11 chloroplast genome sequences were aligned with MAFFT (Katoh and Standley 2013) and then the maximum likelihood (ML) tree was constructed by RAxML (Stamatakis 2014). The results confirmed that *P. tanacetifolia* was clustered with *P. chinensis* (Figure 1).

**Figure 1.**
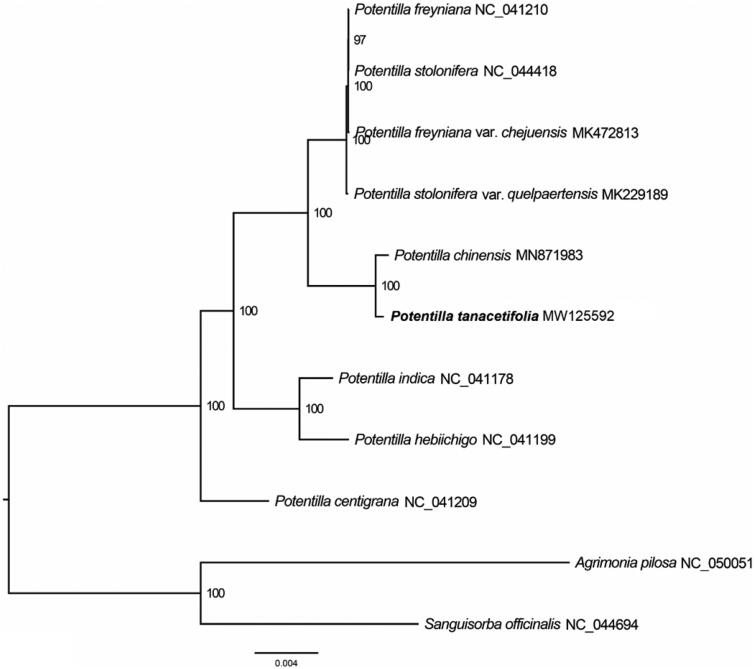
Maximum likelihood (ML) analysis of *P. tanacetifolia* and other related species based on the complete chloroplast genome sequence. Genbank accession numbers: *P. freyniana* (NC_041210), *P. stolonifera* (NC_044418), *P. freyniana* var. *chejuensis* (MK472813), *P. stolonifera* var. *quelpaertensis* (MK229189), *P. chinensis* (MN871983), *P. indica* (NC_041178), *P. hebiichigo* (NC_041199), *P. centigrana* (NC_041209), *Agrimonia pilosa* (NC_050051), and *Sanguisorba officinalis* (NC_044694).

## Data Availability

The genome sequence data that support the findings of this study are openly available in GenBank of NCBI at [https://www.ncbi.nlm.nih.gov] under the accession no. MW125592. The associated BioProject, SRA, and Bio-Sample numbers are PRJNA678424, SRR13062013, and SAMN16796184 respectively.
